# Lactate and pyruvate promote oxidative stress resistance through hormetic ROS signaling

**DOI:** 10.1038/s41419-019-1877-6

**Published:** 2019-09-10

**Authors:** Arnaud Tauffenberger, Hubert Fiumelli, Salam Almustafa, Pierre J. Magistretti

**Affiliations:** 1 Laboratory for Cellular Imaging and Energetics, Biological and Environmental Sciences and Engineering Division, King Abdullah University of Science and Technology, Thuwal, Saudi Arabia; 2 Imam Abdulrahman bin Faisal University, Dammam, Saudi Arabia

**Keywords:** Ageing, Homeostasis

## Abstract

L-lactate was long considered a glycolytic by-product but is now being recognized as a signaling molecule involved in cell survival. In this manuscript, we report the role of L-lactate in stress resistance and cell survival mechanisms using neuroblastoma cells (SH-SY5Y) as well as the *C. elegans* model. We observed that L-lactate promotes cellular defense mechanisms, including Unfolded Protein Response (UPR) and activation of nuclear factor erythroid 2–related factor 2 (NRF2), by promoting a mild Reactive Oxygen Species (ROS) burst. This increase in ROS triggers antioxidant defenses and pro-survival pathways, such as PI3K/AKT and Endoplasmic Reticulum (ER) chaperones. These results contribute to the understanding of the molecular mechanisms involved in beneficial effects of L-lactate, involving mild ROS burst, leading to activation of unfolded protein responses and detoxification mechanisms. We present evidence that this hormetic mechanism induced by L-lactate protects against oxidative stress in vitro and in vivo. This work contributes to the identification of molecular mechanisms, which could serve as targets for future therapeutic approaches for cell protection and aging-related disorders.

## Introduction

Lactate is an important bioenergetic metabolite formed in the absence (fermentation) or presence of oxygen through aerobic glycolysis (Warburg effect) that can be used as an oxidative substrate by cells^1^. Upon its entry into the cell through monocarboxylate transporters (MCTs) and conversion by lactate dehydrogenase (LDH), lactate is oxidized in the mitochondria to produce ATP. Besides its role as an energy substrate, lactate also acts as a gluconeogenic and signaling factor on multiple cell types in different tissues. For instance, lactate promotes transcriptional changes that induce mitochondrial biogenesis^2^. Also, lactate is known to support tumoral cells, which rely on aerobic glycolysis and produce lactate, by promoting cell migration, immune escape, angiogenesis, and expression of pro-survival factors such as HIF1α^3,4^. Similar dual action as an energy substrate and signalling molecule has also been shown in the nervous system^1^. Indeed, neurons, initially believed to rely exclusively on blood-borne glucose, use astrocyte-derived lactate to support their metabolic functions^5^ and to promote synaptic plasticity^1,6–8^. Moreover, the metabolic coupling mechanism known as astrocyte-neuron lactate shuttle (ANLS)^9^ has also established that lactate is protective against different insults including glutamate excitotoxicity and ischemia-reperfusion^10^^,^^11^.

Mitochondria tightly regulate cellular processes involved in energy production and homeostasis, powering ATP production, and producing Reactive Oxygen Species (ROS). Although they are physiological by-products of normal cellular respiration, ROS levels can dramatically increase when the respiratory chain is dysfunctional. Thus, mitochondrial dysfunctions during aging have been associated with cellular stress and neurodegenerative diseases, including Alzheimer’s and Parkinson’s diseases^12^. The energetic deficits during aging are also correlated with the loss of proteostasis, a set of processes controlling the homeostasis of the biogenesis, folding, trafficking, and protein degradation^13,14^.

Until recently, ROS were considered primarily as a hallmark of oxidative stress leading to cellular dysfunction and neurodegeneration^15,16^. Growing evidence now indicates that ROS may also act as signaling molecules in physiological processes. Indeed, exposure for short periods or low concentrations of ROS contributes to increased lifespan in multiple organisms^17–19^ in a pro-survival mechanism called hormesis or mitohormesis^20–22^. Here we show that lactate promotes resistance to oxidative stress in both mammalian cells and *C. elegans*, where pyruvate reproduces the protective effects mediated by lactate. We found that lactate supplementation induces a moderate elevation in ROS levels and the transcription of genes belonging to pro-survival pathways, including the IGF-AKT/PI3K and the endoplasmic reticulum stress pathways. Our observations suggest that lactate, and to a lesser extent pyruvate, supports resistance to cellular stress through a mild hormetic increase in oxidative stress.

## Methods

### Cell culture

SH-SY5Y neuroblastoma cells were grown in DMEM-F12 media (*Gibco - #11320-074*) supplemented with 10% FBS (*Gibco - #3000008085*) and 1% Pen/Strep mix (*Gibco - #15140122*), at 37 °C with 5% CO2. Cells were maintained in T75 flasks, and media was changed every 2 days. For the experiments, cells were subcultured in different dish formats (6/24 wells plates) and used 36 h after seeding. Sodium L-lactate (*Sigma - #71718*) and sodium pyruvate (*Sigma - #P2256*) were used in this study to investigate the role of both metabolites in cell survival.

For pharmacological treatments, inhibitors (**AR-C155858** – *Tocris #4960*, **LY294002** – *Sigma #L9908*, **Quercetin** – *Sigma #00200595*, **Cycloheximide** – *Sigma #C1988* and **N-acetylcysteine** – *Sigma #A7250*) were applied 15 min before lactate or pyruvate stimulation, and all treatments were conducted in DMEM-F12 media supplemented with 10% FBS.

### Cell viability assays

Cells were seeded 36 h prior to assays to maximize their recovery and to allow a confluence of around 75–80% at the time of treatment. Cells were treated with lactate or pyruvate for 6 h in DMEM-F12 + 10% FBS. The media was then replaced with DMEM-F12 containing fresh H_2_O_2_ at a final concentration of 150 μM. The next day the media was collected, and cells trypsinized for analysis. The percentage of cells excluding the trypan blue stain was determined using a Countess II FL Automated Cell Counter (ThermoFisher) according to the user manual’s instructions.

For the MTT assay, MTT (3-[4,5-dimethylthiazol-2-yl]-2,5 diphenyl tetrazolium bromide) was added to cell media at a final concentration of 0.2 mg/ml. Cells were treated for 2 h to allow the transformation to formazan. After incubation, media was replaced by DMSO to dissolve the crystals, and absorbance was measured at λ = 570 nm.

### ROS detection

Cells were treated in under different conditions: (1) 6 h with 20 mM lactate in the presence or absence of 0.1 mM N-acetylcysteine or (2) with 150 μM H_2_O_2_ for 30 min or (3) 6 h with 20 mM pyruvate. After the initial treatments, 50 μM 2′,7′-dichlorodihydrofluorescein diacetate (H_2_DCFDA) was added to the media for 30 min. Cells were washed in HBSS media (136 mM NaCl, 3 mM KCl, 1,25 mM CaCl_2_, 1,25 mM MgSO_4_, 10 mM HEPES and 2 mM D-glucose) and ROS levels were measured.

Nematodes were treated with 100 μM H_2_DCFDA for a 1 h, washed 3 times with M9 buffer (3 g/L KH_2_PO_4_, 6 g/L Na_2_HPO_4_, 5 g/L NaCl, and 1 mM MgSO_4_) and scored on a 2% agarose pad with an M9 buffer with 5 mM Levamisole. Both cells and worms ROS levels were measured using a Zeiss LSM780 and AxioExaminer at λ = 530 nm.

### JC1 staining

Cells were treated with different conditions: 6 h with 20 mM lactate or with 150 μM H_2_O_2_ for 30 min. After the initial treatments, 2.5 μM of JC-1 staining was added to the media for 10 min. Cells were washed in HBSS media (136 mM NaCl, 3 mM KCl, 1,25 mM CaCl_2_, 1,25 mM MgSO_4_, 10 mM HEPES and 2 mM D-glucose) and fluorescence levels were measured using a Zeiss LSM780 and AxioExaminer at λ = 530 nm and 590 nm.

### RNA extraction

Total RNA from SH-SY5Y cells was isolated using the RNeasy Plus Mini Kit (Qiagen) following the manufacturer’s instruction.

### RNA sequencing methods

Concentration, purity, and integrity of the RNA extracted from the neuroblastoma cells were assessed with a NanoDrop spectrophotometer (NanoDrop 2000, ThermoFisher Scientific), and a 2100 Bioanalyzer (Agilent).

Total RNA with an RNA Integrity Number above 9.5 was used to construct libraries using the TruSeq Stranded mRNA Sample Kit (Illumina) following the protocol’s instructions. Briefly, mRNA was enriched using oligo dT-attached magnetic beads, fragmented, and converted into cDNA. Fragments of cDNA went through an end repair process, 3′ ends were adenylated, universal bar-coded adapters were ligated, and cDNA fragments were amplified by PCR to yield the final libraries. The sequencing libraries were evaluated using a 2100 Bioanalyzer (Agilent). Paired-end read (2 × 150 bp) multiplex sequencing from pooled libraries was performed on an Illumina HiSeq 4000 machine at the KAUST Bioscience Core Labs. An average of 40–50 million reads was obtained for each sample. Sequencing data have been deposited in the NCBI SRA database under the project accession number PRJNA510906.

Raw read quality was evaluated with the FastQC tool (https://www.bioinformatics.babraham.ac.uk/projects/fastqc/). Low-quality reads were filtered out and adapter sequences trimmed using Trimmomatic version 0.36^23^ with the following parameters: ILLUMINACLIP/TruSeq3-PE-2.fa:2:30:10, LEADING:3, TRAILING:3, SLIDINGWINDOW:4:15, MINLEN:36. Reads from each sample replicate were mapped to the human reference genome (Ensembl, release 91) using STAR version 2.6.0a^24^ with default parameters except for outFilterMultimapNmax set to 1 (using Hisat2 version 2.1.0^25^ with default parameters except for k set to 1). Mapped reads for protein-expressing genes were summarized with the featureCounts program (Subread package, version 1.5.2)^26^, and the differential expression analysis was performed with the Bioconductor package DESeq2^27^ in the R programming environment. To minimise background noise and to focus on more significant genes in term of biological impact, we removed genes with very low expression levels, excluding genes that failed to total an average count above 10 in any conditions. Differentially expressed genes (DEG) were considered in pairwise comparisons with a threshold including a fold change expression ≥ 1.5 and q-value (or False Discovery Rate, FDR) < 0.05. To obtain a functional representation of the lists of DEG, we performed gene ontology (GO) and Biocarta and KEGG pathways enrichment analyses using the online database and tool DAVID (version 6.8, https://david.ncifcrf.gov).

### Transcription factor binding site enrichment analysis

Gene ID were used in transcription factor enrichment analysis, and the analysis was run with default parameters for *Homo sapiens* using the UCSC_TFBS tool on DAVID online database (version 6.8, https://david.ncifcrf.gov)

### ***C. elegans***

Standard methods of culturing and handling worms were used. Worms were maintained on standard NGM plates streaked with OP50 Escherichia coli, and all strains were scored at 20 °C unless indicated. Mutant strains were obtained from the *C. elegans* Genetics Center (University of Minnesota, Minneapolis, MN, USA). Mutants or transgenic worms were verified by visible phenotypes, PCR analysis for deletion mutants, sequencing for point mutations, or a combination thereof. Deletion mutants were out-crossed a minimum of three times to wild-type worms before use. The strains list can be found in Table S1.

### Measure of aging phenotypes in *C. elegans*

#### Juglone (oxidative stress) test

Worms were grown on NGM (OP50-1) in the presence or absence of 100 mM lactate or pyruvate until and then transferred to NGM containing 300 μM juglone (5-Hydroxy-1,4-naphthoquinone, Sigma). Juglone was dissolved in 96% ethanol.

#### Lifespan assay

Nematodes were grown on NGM supplemented or not with lactate or pyruvate. Adult day 1 one animals, 30 per plates in triplicates, were transferred onto NGM supplemented with 20 μM FUdR, and lifespan was assessed at 20 °C. Animals were declared dead if they failed to respond to tactile or heat stimuli. For dead bacteria experiments, OP50-1 bacteria were heat-killed by incubating the culture at 80 °C for 4 h.

### Immunoblotting

SH-SY5Y cells were harvested from six-wells plate and washed with cold 1X PBS. *C. elegans* were collected in M9 buffer, and the pellets were quickly frozen at −80 °C overnight. Both tissues were lysed using RIPA buffer (150 mM NaCl, 50 mM Tris pH 7.4, 1% Triton X-100, 0.1% SDS, 1% sodium deoxycholate) containing 1X Protease and Phosphatase Inhibitor Cocktail (*ThermoFisher #78440*). Protein concentration was measured using Bicinchoninic assay (BCA – Thermofisher). Protein extracts were then loaded on 10% SDS-page acrylamide gels and transferred on PVDF membrane *(Millipore #IPVH00010)* overnight. The membranes were blocked using in PBS containing 0.1%Tween and 5% milk for at least 30 min. Primary antibodies were applied overnight at 4 °C, and subsequently, HRP-conjugated secondary antibodies were incubated for 1 h before visualisation using ECL (*ThermoFisher #34096*). hif-1α [EP1215Y] (abcam – ab51608) and GRP-78/Bip (abcam – ab32618).

### Quantification and statistical analysis

For Trypan blue exclusion test, each data point represents one measurement. Bars are the average ± SEM. All calculations for statistical significance were completed using a non-parametric, one-way ANOVA with multiple comparisons, three replicates/independent experiment were measured in a minimum of four independent experiments.

Survival curves were analyzed using the Log-Rank (Mantel-Cox) test. 90 animals/trial **(lifespan)** or 50 animals/trial **(Juglone)** were tested, in a minimum of three independent experiments. See Table S2 for lifespan statistics.

For the quantitative GFP summaries, each data point represents the mean fluorescence value of one animal. Horizontal bars are the average ± SEM. Statistical significance (*p < 0.05, **p < 0.01, ***p < 0.001, and ****p < 0.0001) was calculated using a non-parametric, one-way ANOVA with multiple comparison tests. 30–40 animals/trial were tested in a minimum of 3 independent experiments.

GraphPad Prism 7 was used for statistical analysis and ImageJ to quantify the images and the. Experiments were performed in triplicate.

## Results

### Lactate pre-treatment reduces cell death induced by oxidative stress

**Fig. 1:**
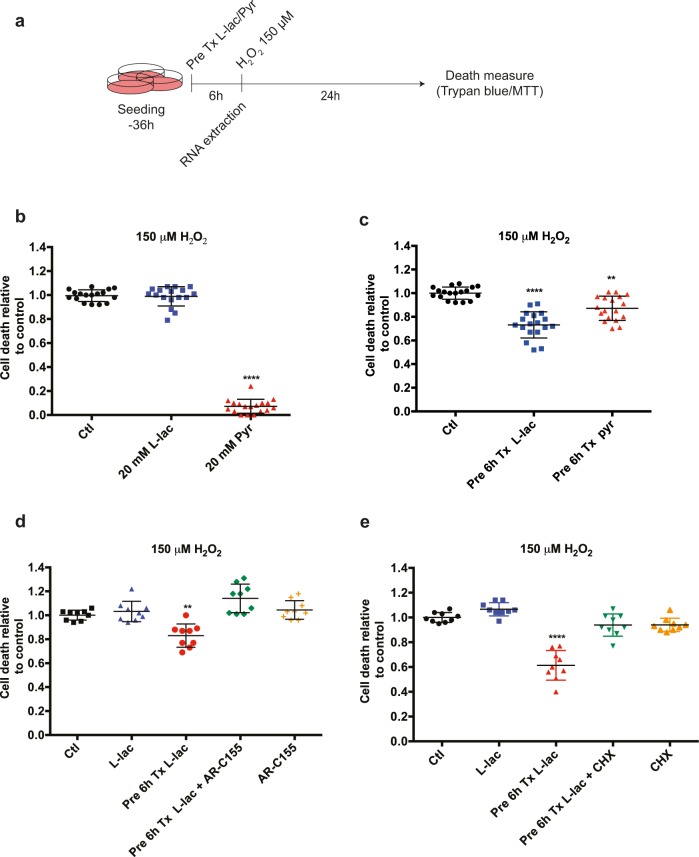
Pre-treatment with lactate increases resistance to oxidative stress. **a** Schematic representation of the experimental procedures used in this study with the SH-SY5Y cells. **b** Measures of cell death after co-application of 150 μM H_2_O_2_ with lactate or pyruvate. **c** Measures of cell death after oxidative stress induced by 150 μM H_2_O_2_ following a 6 h pre-treatment with lactate or pyruvate. **d**, **e** Measures of cell death after oxidative stress induced by 150 μM H_2_O_2_ upon pre-treatment with lactate or pyruvate and application of 1 μM MCT1 inhibitor AR-C155858 (**d**) or 1 μM cycloheximide (**e**). Each data point represents one measurement. Bars are the average ± SEM. All calculations for statistical significance were completed using a non-parametric, one-way ANOVA with multiple comparisons, *n* = 12–15 from 4–5 independent experiments. **p < 0.01, ****p < 0.0001

Lactate has been shown to play a role in enhancing neuronal survival^11,28^. These studies indicated that lactate promotes an increased ATP production and a better Ca^2+^ buffering in a model of excitotoxicity. We, therefore, set out to investigate whether lactate could also promote cell protection using SH-SY5Y neuroblastoma cells, a widely used model of cell toxicity^29^ and lactate-mediated physiological effects^30^. We treated the cells with a toxic concentration of hydrogen peroxide (H_2_O_2_), one of the primary cellular ROS, and assessed cell death using the Trypan Blue exclusion method (Fig. 1a). Initial results showed that a concentration of 150 µM of H_2_O_2_ resulted in 70–80% of cell death after 24 h of oxidative stress. Under these conditions, we tested whether supplementing the medium with lactate (20 mM) could promote cell survival. Co-application of lactate together with H_2_O_2_ did not counteract the toxic effects of H_2_O_2_-induced oxidative stress, while pyruvate co-treatment massively reduced cell death. The latter observation results from direct chelation of H_2_O_2_ by pyruvate, leading to its inactivation in the cell culture media^31,32^ and was considered as artefactual (Fig. 1b).

We next tested a possible effect of lactate and pyruvate as pre-treatment on cell death triggered by H_2_O_2_ exposure. Lactate media supplementation for 6 h prior to oxidative stress exposure resulted in significantly increased survival (26,8%) **(**Fig. 1c and S1a) while a more modest effect was observed with pyruvate. These observations were not dependent on the detection method for cell death, as MTT assay offered comparable results to Trypan Blue exclusion (Fig. S1b). An osmolarity effect can be excluded as pre-treatment with 20 mM NaCl, D-glucose, or Na-gluconate did not affect cell survival upon H_2_O_2_ treatment (Fig. S1c). Finally, lactate but not pyruvate was also able to rescue cell death upon treatment with two other oxidative stress inducers, tert-butyl hydroperoxide and sodium arsenite (Fig. S1d, e), suggesting that the observed protective effect is not limited against the stress triggered by H_2_O_2_. Taken together, these results suggest that pre-treatment of cells with lactate is effective in protecting cells from oxidative stress.

Lactate and pyruvate are transported through cells via a family of plasma membrane monocarboxylate transporters (MCT). We examined the lactate-dependent decrease in H_2_O_2_-induced cell death in the presence of the MCT blocker AR-C155858. Inhibition of MCT was found to decrease the protective effect of lactate (Fig. 1d) on H_2_O_2_-evoked oxidative stress. Similar attenuation of lactate’s effect was also observed using UK5099, another MCT inhibitor (Fig. S1f). These data indicate that the entry of lactate into cells is necessary for its protective effects on oxidative stress-induced cell death.

We also established that lactate enhanced stress resistance through de novo protein synthesis, using the translation blocker cycloheximide (Fig. 1e).

### Lactate reduces cell death through induction of pro-survival and proteostatic pathways

**Fig. 2:**
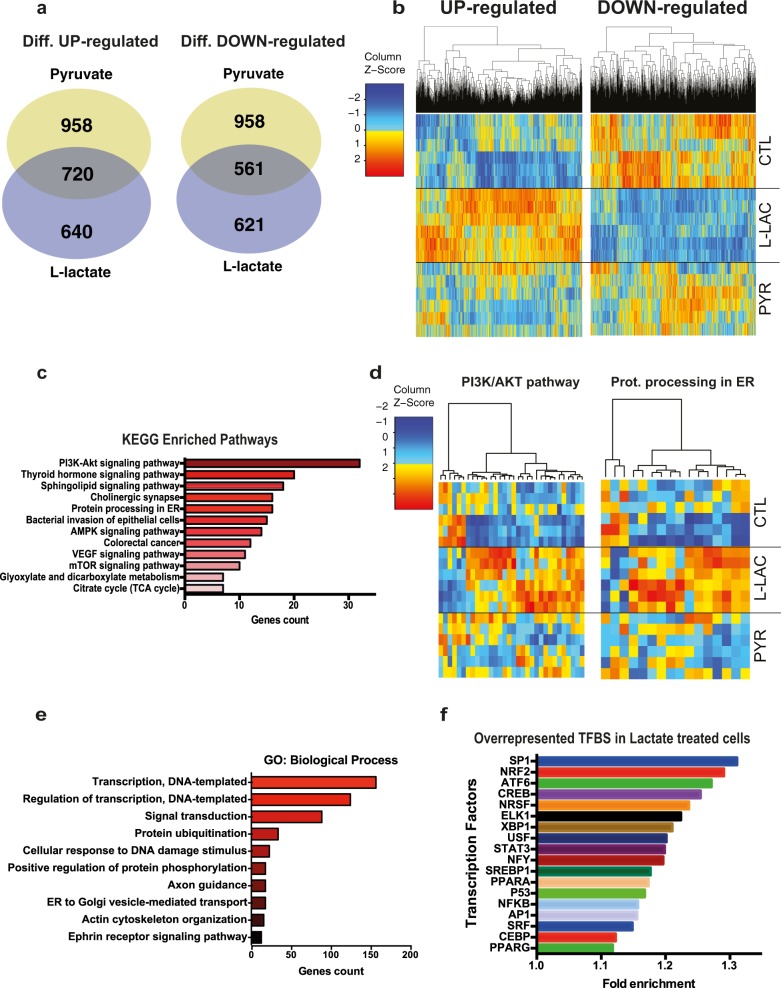
RNA sequencing of SH-SY5Y cells revealed enrichment in key growth-survival pathways. **a** Venn diagram showing the overlap between the numbers of significant differentially up- and down-regulated genes compared to control by lactate and pyruvate. SH-SY5Y cells were treated for 6 h with 20 mM lactate or pyruvate. The cut-off for significance was *p* < 0.01. **b** Heat map clustering of genes differentially regulated by lactate, compared to control and pyruvate-treated cells. The map represents expression levels of each transcript (count per million), and z-score represents a normalized expression of each transcript across the different conditions. **c** KEGG pathways over-representation analyses. Significantly enriched pathways (*p*-value < 0.05) are ranked by genes count **d** Heatmap clustering for KEGG-PI3K/AKT and KEGG-Protein processing in ER. Each map represents the list of genes specifically regulated by lactate and associated with PI3K/AKT or Protein processing in the ER. The map represents expression levels of each transcript (count per million), and z-score represents a normalized expression of each transcript across the different conditions. **e** Biological processes GO terms ranked by genes count (*p*-value < 0.05). **f** Sublist of DAVID over-representation of Transcription factors binding sites curated for metabolism and stress resistance signaling, associated with differentially expressed genes by lactate. Significantly enriched transcription factors are ranked by fold enrichment

**Fig. 3:**
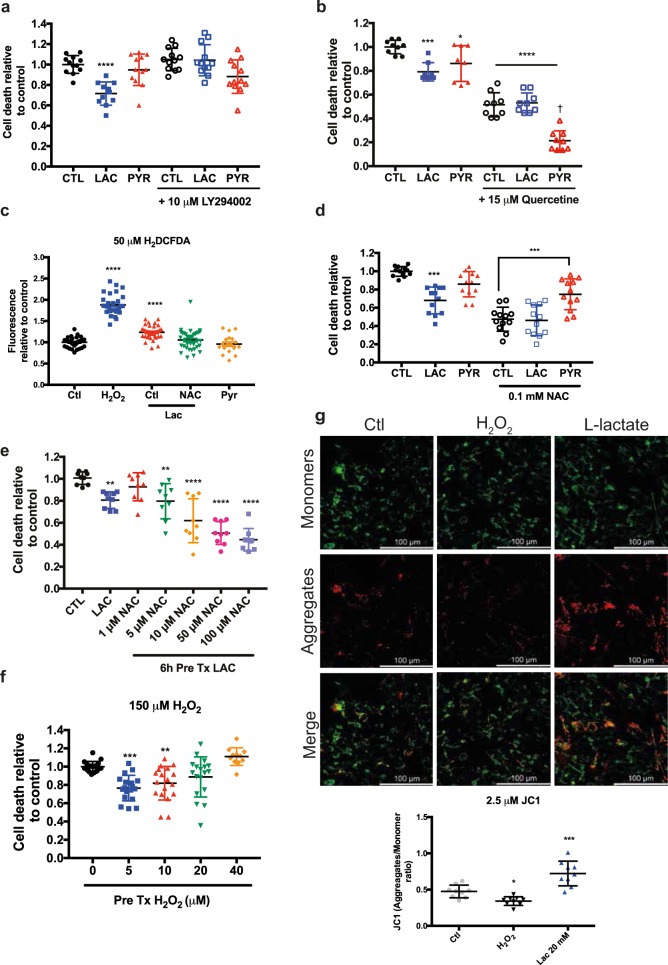
PI3K and UPR^ER^ pathways mediate lactate-mediated cell survival through ROS production. **a** Relative cell death assessed by trypan blue exclusion following oxidative stress induced by 150 μM H_2_O_2._ Prior to oxidative stress exposure, SH-SY5Y cells were co-treated for 6 h with 10 μM LY2940002 and lactate or pyruvate. **b** The measure of cell death after 150 μM H_2_O_2_ treatment upon pre-treatment with lactate and pyruvate. Cells were co-treated with UPR^ER^ inhibitor (Quercetine – 15 μM) with 20 mM lactate or pyruvate. **c** The measure of ROS level using H_2_DCFDA upon 150 μM H_2_O_2_, 20 mM lactate or pyruvate and 100 μM N-acetylcysteine. **d** Relative cell death measurements following oxidative stress induced by 150 μM H_2_O_2._ Prior to oxidative stress exposure, cells were co-treated for 6 h with 100 μM N-acetyl-cysteine lactate or pyruvate. **e**. Relative cell death measurements following oxidative stress induced by 150 μM H_2_O_2._ Prior to oxidative stress exposure, cells were co-treated for 6 h with increasing doses of N-acetyl-cysteine and 20 mM lactate. **f** Measure of cell death after 150 μM H_2_O_2_ and pre-treated with low doses of H_2_O_2_. **g** Confocal images of SH-SY5Y cells stained with JC1 (2.5 μM). Cells were treated with 150 μM H_2_O_2_ for 30 min or with 20 mM lactate for 6 h. (*N* *=* *4*), *p < 0.05, **p < 0.01, ***p < 0.001, ****p < 0.0001, ^†^(*p* < 0.0001 compared to Quercetine treated ctl)

To further characterize the protective effect of lactate, we analyzed the expression of over 13000 genes using whole transcriptome RNA sequencing (RNAseq). Treatment with lactate affected the expression of 1261 (640 upregulated and 621 downregulated) (Table S3) compared to control and pyruvate-treated cells (Fig. 2a, b). Over-representation analysis of KEGG pathways for the genes regulated by lactate revealed the involvement of PI3K, mTOR signaling, and protein processing in ER pathways (Fig. 2c, d). Gene ontology analysis for biological processes also revealed enrichment for terms related to cell survival processes such as protein ubiquitination and response to DNA damage (Fig. 2e). Finally, our sequencing data also showed that within the promoter region of the genes differentially regulated by lactate, we observe an over-representation of binding sites for transcription factors involved in protein homeostasis in ER (ATF6 and XBP1) and ROS detoxification (NRF2) (Fig. 2f and Table S4). These observations are consistent with previous findings suggesting that lactate-mediated neuroprotection involves the PI3K signaling pathway^11^. mTOR signaling is a major pathway involved in cell growth and survival in different cell types^33,34^. Among the genes specifically affected by lactate, we identified TSC2 and S6K (mTOR signaling), AKT1 (PI3K pathway), as well as ATF4, GRP78/BiP and XBP1 (ER processing) (Fig. S2a–f). GRP78/BiP and XBP1 are part of the unfolded protein response in the ER (UPR^ER^) and promote protein homeostasis^35,36^. Additionally, we identified several ER chaperones uniquely regulated by lactate, including HSP2A, DNAJA2, DNAJC5, and DNAJC10 (Fig. S2g–j). To validate these important pathways in the protective effects induced by lactate, we first examined the role of the PI3K/Akt pathway by treating the cells with a potent PI3K inhibitor. Co-application of lactate and LY294002 blocked the rescue by lactate of oxidative stress-evoked cell death (Fig. 3a). The contribution of the UPR^ER^ in the lactate-dependent protection against ROS toxicity was examined using quercetin, a flavonol known to reduce UPR^ER^ activity^37^. Application of quercetin prevented the protective effect exerted by lactate on H_2_O_2_-mediated cell death (Fig. 3b). This observation suggests that activation of a mild ER stress is required for lactate-mediated oxidative stress resistance. Taken together, these results suggest that lactate promotes cell survival against oxidative stress through the induction of key homeostatic pathways that include PI3K, mTOR, and ER protein processing.

### Lactate reduces cell death through ROS signaling

Interestingly, most of the pathways described in the preceding section have been linked to ROS signaling. Detailed examination of the transcriptomics data, focusing on ROS detoxification enzymes, provided evidence that the expression of HIFα transcript is increased by lactate (Fig. S2k) while that of NRF2 was increased by both lactate and pyruvate pre-treatment (Fig. S2l). The effectiveness of a pre-treatment as a protective mechanism suggests the induction of a protective response or hormesis that would reduce the impact of lethal stress later on^20,21,38^. Work by others has shown that lactate promotes ROS induction^39,40^, suggesting that this mechanism could mediate the observations reported here.

We measured ROS production in cells pre-treated with lactate or pyruvate using 2′,7′-dichlorodihydrofluorescein di-acetate (H_2_DCFDA), a fluorescent dye detecting reactive oxygen intermediates. ROS production was increased after exposure to lactate for 6 h, compared to both control and pyruvate-treated cells. This increase was dose-dependent (Fig. S3a) and lost upon treatment with antioxidant N-acetyl-L-cysteine (NAC, 0.1 mM) (Fig. 3c and Fig. S1g)^41^. In the presence of NAC, lactate was not able to reduce cell death further, indicating that cell protection evoked by lactate requires an increase in ROS levels (Fig. 3d). NAC decreased by itself the overall cell death upon H_2_O_2_ treatment, but we confirmed our hypothesis by adding increasing concentrations of NAC to cells pre-treated with lactate. A low concentration of NAC (1 μM), which was not able on its own to reduce overall cell death by H_2_O_2_, nevertheless blocked the protective effect of lactate (Fig. 3e). Furthermore, low doses of H_2_O_2_ mimicked lactate-mediated protection against oxidative stress (Fig. 3f). These data confirm that ROS induction by lactate is required for cell survival upon oxidative stress.

We next examine if lactate could affect mitochondrial function. Using staining with JC1, a fluorescent mitochondria indicator forming red-shifted J-aggregates at high membrane potentials^42^, we observed an increase in mitochondrial potential (red aggregates) upon lactate treatment (Fig. 3g). In contrast, high doses of H_2_O_2_ dramatically reduced mitochondrial membrane potential (green staining). These results indicate that lactate increases mitochondrial respiration resulting in a mild induction of ROS levels, while a high H_2_O_2_ concentration disrupts membrane potential, causing mitochondrial dysfunction and cell death. Inhibition of NADPH oxidase, a source of cytoplasmic ROS, did not prevent lactate-mediated stress resistance, suggesting that this enzyme complex is not required for the reported effects (Fig. S1h). Besides the regulation of transcription factors NRF2 and HIF1α expression, the RNAseq analysis did not reveal changes in the expression of effector enzymes controlling ROS detoxification after 6 h. However, a measure of the expression of SOD1, PRDX5, GSTM4, and GPX3 3 h after the end of exposure to lactate or pyruvate showed a significant increase. (Fig. S2m–p). Altogether, the effects reported here strongly suggest that lactate supplementation induces a mild ROS production, which in turn activates pro-survival pathways including mTOR, PI3K, and ER protein processing.

### Lactate and pyruvate delay aging-evoked phenotypes in *C. elegans*

**Fig. 4:**
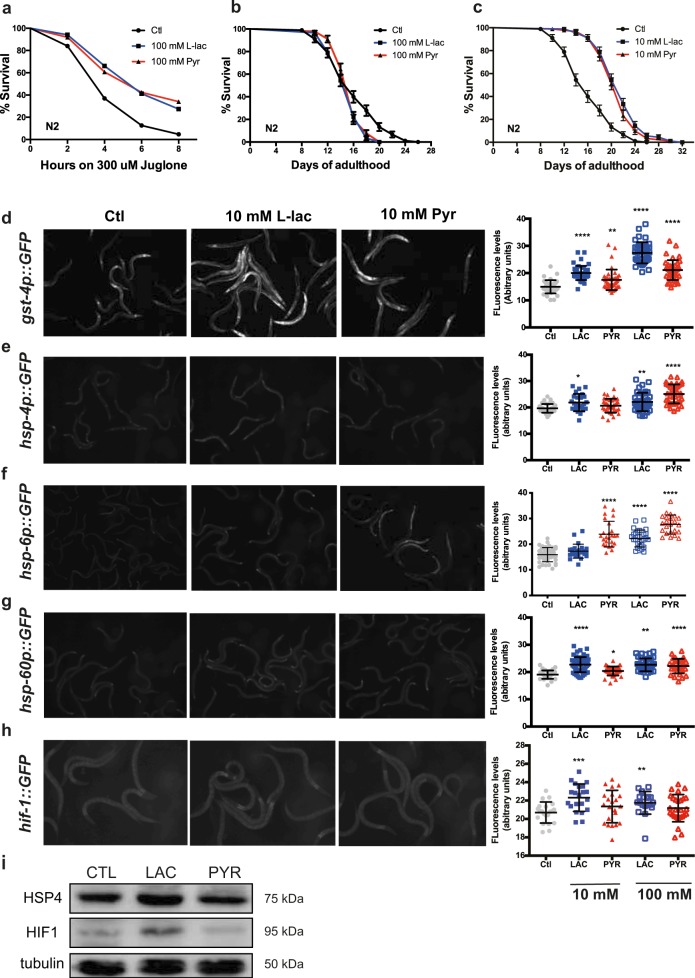
Lactate and pyruvate promote stress resistance and induce cellular stress response. **a** Survival curves of wild-type nematodes supplemented with lactate or pyruvate treated with 300 μM juglone (*p* < 0.0001) **b**, **c** Lifespan curves of wild-type animals treated with 100 mM (**b**) or 10 mM (**c)** lactate or pyruvate. **d**–**h** Representative GFP fluorescence images and quantitative measurements of GFP fluorescence intensities in *C.elegans* expressing transcriptional reporters, as indicated in the left margin, treated with 10 mM lactate or pyruvate. **i** Immunoblot analysis of HIF1α and HSP4/GRP78 protein expression in wild-type nematode. Tubulin is used as a loading control. (*N* *=* *3*, *p < 0.05, **p < 0.01, ***p < 0.001, and ****p < 0.0001)

We next took advantage of *C. elegans’s* simple genetics and short lifespan to investigate, in vivo, the role of lactate in stress resistance and longevity. First, we supplemented their bacterial diet with lactate or pyruvate and induced oxidative stress by exposure to the natural compound juglone^43^. Animals supplemented in their diet with high concentrations of lactate or pyruvate (100 mM) were more resistant to juglone than control nematodes (Fig. 4a) but lifespan of wild-type animals was reduced (Fig. 4b). Lower concentrations of lactate or pyruvate (10 and 50 mM) did not increase survival, suggesting that the protective mechanisms underlying the stress resistance were not triggered (Fig. S3b). However, 10 mM of lactate or pyruvate-increased lifespan of WT nematode (Fig. 4c). The increased stress resistance was not caused by secondary metabolism of lactate or pyruvate by the nematodes’ bacterial food source; animals grown on heat-killed bacteria with no metabolic activity showed the same response to lactate and pyruvate (Fig. S3b).

### Lactate and pyruvate induce cellular defence mechanisms in *C. elegans*

Next, we investigated several well-described transcriptional/translational reporters’ induction by lactate and pyruvate in the worm to determine how the monocarboxylates promote cell survival and longevity. The expression glutathione-S-transferase (*gst-4*), a downstream target of the transcription factor NRF2/*skn-1* known to contribute to cellular homeostasis against ROS and ER stress^44^, was strongly induced in animals supplemented with lactate or pyruvate (Fig. 4d). Both metabolites also induced the expression of the ER (*hsp-4*) and mitochondrial (*hsp-6* and *hsp-60*), stress chaperones (Fig. 4e–g). Lactate was also able to induce hypoxia-inducible factor *hif-1* reporters (Fig. 4h). However, expression of *daf-16*, *sod-3*, *lgg-1*, and *crtc-1* reporters was not influenced by lactate or pyruvate supplementation (Fig. S3c–f). These results indicate that pathways involved in mammalian cell protection, including ROS detoxification mechanisms, HIF1α, and ER stress responses, identified in SH-SY5Y cells, are activated by lactate or pyruvate in the nematode. Additionally, we determined that the mitochondrial unfolded protein response (UPR^mt^) is induced by lactate and pyruvate supplementation in the nematode.

### Lactate and pyruvate promote oxidative stress resistance via UPR and p38MAPK pathways

**Fig. 5:**
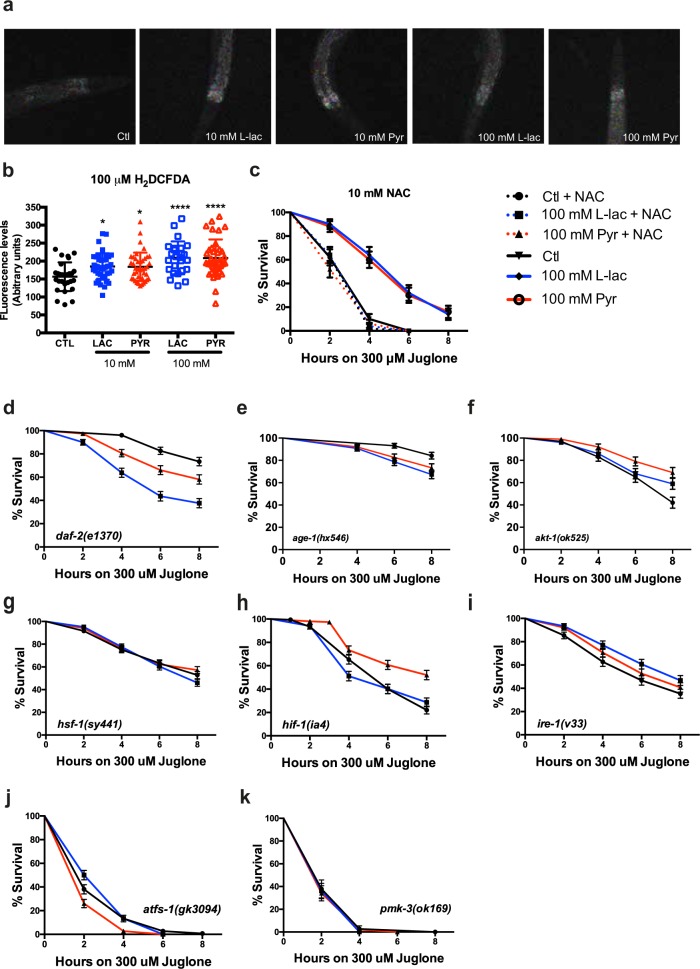
Lactate and pyruvate increase stress resistance through ROS induction. **a**, **b** Representative H_2_DCFDA fluorescence images **a**) and quantitative measurements of H_2_DCFDA fluorescence intensities **b**) in wild-type nematodes supplemented with 10 mM or 100 mM lactate or pyruvate. **c** Survival curves of wild-type nematodes treated with 300 μM juglone and supplemented with 10 mM N-acetyl cysteine (NAC) together with 100 mM lactate or pyruvate. **d**–**k** Survival curves of mutant nematodes treated 300 μM juglone and supplemented with 100 mM lactate or pyruvate. Mutated genes are indicated as an inset in the lower left or right corner of each graph. (*N* *=* *3*, *p < 0.05 and ***p < 0.001)

Following on our observations in SH-SY5Y cells and the observation of increased activity of *gst-4* in the worm (Fig. 4d), we assessed the role of ROS production upon lactate and pyruvate in stress response in the nematode. Similar to our findings on SH-SY5Y cells (Fig. 3c), Lactate and pyruvate induced ROS production in a dose-dependent manner, compared to untreated controls (Fig. 5a, b, Fig. S3g), and the use of 10 mM NAC on worms supplemented with lactate or pyruvate abolished the resistance to juglone (Fig. 5c).

To understand how ROS induction promotes oxidative stress resistance, we performed a mutant screen with candidate genes. We chose a broad array of mutant animals with defective metabolism, stress response, and transcriptional regulation pathways. We exposed the mutant animals supplemented with lactate or pyruvate to juglone. If the monocarboxylates were protective in the absence of a given signaling pathway, then that pathway is unlikely to be the cause of protection.

The Insulin/IGF signaling (IIS) pathway is a well-conserved longevity pathway that responds to metabolic changes and triggers the activation of pro-survival mechanisms^45^. Three components of the (IIS) appeared to be involved in the stress resistance mediated by both monocarboxylates: The knockdown of IGF receptor *daf-2* and the kinases *age-1* and *akt-1* limited stress resistance by lactate or pyruvate (Fig. 5d–f). Interestingly, *akt-1* was also identified in our screen in SH-SY5Y cells. Surprisingly, the canonical downstream transcription factor of this pathway, *daf-16*, did not influence lactate- or pyruvate-mediated resistance to juglone (Fig. S4a). The loss *hsf-1*, a known regulator of stress resistance^46^ and downstream target of *daf-2*, also abolished the stress resistance (Fig. 5g). These observations are in line with previous studies showing that HSF1 and HIF1ɑ are affected by the redox state of cells and the oxidation of cysteines residues by reactive species^47^.

Hypoxia-inducible factor 1 (*hif-1*) regulates resistance to hypoxia and longevity upon ROS induction^48,49^. Our mutant screen revealed that a knockdown of *hif-1* blocked the protective effect by lactate and reduced the effect of pyruvate upon oxidative stress (Fig. 5h). This is consistent with our observation that *hif-1* is upregulated upon supplementation with lactate **(**Fig. 4h**)**.

We also observed that *ire-1, and atfs-1* mutants, involved respectively in the unfolded protein response in ER and the mitochondria^50,51^, blocked lactate- or pyruvate-evoked stress resistance (Fig. 5i, j). *ire-1* expression was also enriched in our transcriptomic analysis in SH-SY5Y cells, and it is implicated in NRF2-mediated ROS detoxification processes^44,52^. However, although the loss of *atfs-1* prevented increase resistance to juglone, the electrons transport chain (ETC) disruption by complex I and III mutants, *nuo-6* and *isp-1*^19,53^ did not influence survival increase evoked by lactate and pyruvate under juglone treatment (Fig. S4b, c)

More surprisingly, knockdown p38 MAPK ortholog *pmk-3* also blocked stress resistance by lactate and pyruvate (Fig. 5k). *pmk-3* is known to regulate longevity induced by mitochondrial disruption and axonal regeneration pathways^54,55^. However, knockdown of the *pmk-3* canonical upstream control *dlk-1* did not influence lactate- or pyruvate-mediated stress resistance (Fig. S4d).

Our mutant screen also showed that well-known genes for canonical pathways involved in metabolism, survival, and cellular signaling did not influence the protective effects elicited by lactate or pyruvate (Fig. S4e–o).

Overall, consistent with our work on SH-SY5Y cells, lactate and pyruvate also stimulate stress resistance through ROS production in the worm. Furthermore, similar survival pathways mediate this protection.

### *ire-1* and *pmk-3* mediate *gst-4* induction by lactate and pyruvate

**Fig. 6:**
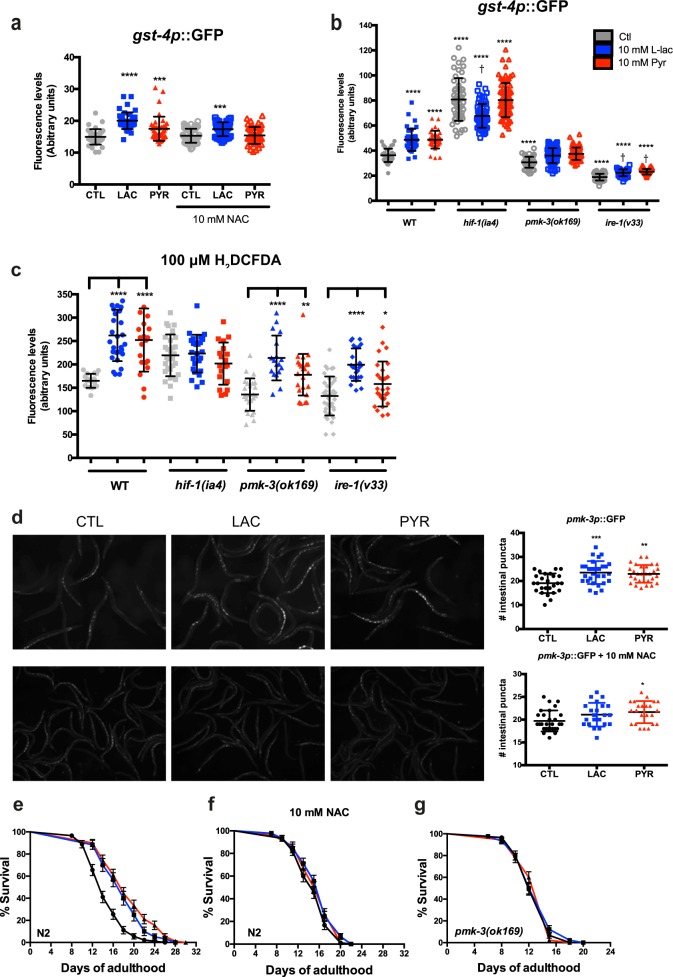
i*re-1* and *pmk-3* mediate *gst-4* induction by lactate and pyruvate. **a** Quantitative summaries of GFP fluorescence in nematodes expressing the transcriptional reporter *gst-4*p::GFP supplemented with 10 mM lactate or pyruvate. Animals were co-treated with 10mM N-acetylcysteine (NAC) and 10 mM lactate or pyruvate. **b** Quantitative summaries of GFP fluorescence in nematodes expressing the transcriptional reporter *gst-4*p::GFP supplemented with 10 mM lactate or pyruvate in *hif-1(ia4)*, *pmk-3(ok169)* and *ire-1(v33)* mutant backgrounds. **c** Quantitative summaries of GFP fluorescence in nematodes treated with 100 μM H_2_DCFDA supplemented with 10 mM lactate or pyruvate in *hif-1(ia4)*, *pmk-3(ok169)* and *ire-1(v33)* mutant backgrounds. **d** Quantitative summaries of GFP fluorescence in nematodes expressing the *pmk-3*p::*pmk-3*::GFP reporter supplemented with 10 mM lactate or pyruvate on regular NGM or supplemented with 10 mM NAC. **e**, **f** Survival curves for N2 animals treated with 10 mM lactate or pyruvate on regular NGM (**e**) or supplemented with 10 mM NAC (**f**). **g** Survival curve for *pmk-3(ok169)* supplemented with 10 mM lactate or pyruvate. (*N* *=* *3*, *p < 0.05, **p < 0.01, ***p < 0.001, and ****p < 0.0001

We examined whether our candidates (*hif-1*, *ire-1* and *pmk-3*) could influence the expression of the gst-4 reporter. Co-treatment of wild-type worms with NAC blocked the activation of *gst-4* induced by lactate or pyruvate supplementation (Fig. 6a). Although mutation in *hif-1* surprisingly increased *gst-4* activity above control levels, both metabolites did not further increase the levels of the reporter (Fig. 6b, Fig. S5a–c). Loss of *pmk-3* **(**Fig. 6b, Fig. S5d–f) and *ire-1* (Fig. 6b, Fig. S5g–i) reduced the basal activity of *gst-4*. Supplementation with lactate or pyruvate, although marginally increasing the reporter expression in *ire-1* mutants, failed to restore the *gst-4* activity to wild-type levels. A measure of ROS in *hif-1* mutants confirmed an increase in ROS concentration while lactate or pyruvate were not able to further induce accumulation of reactive species. However, *pmk-3* and *ire-1* mutants were not different from N2 animals, and both lactate and pyruvate induced a ROS burst (Fig. 6c). Using a translational reporter of *pmk-3*, we observed that lactate and pyruvate induced expression the p38 MAPK and that this effect was prevented by supplementation of the media with 10 mM NAC (Fig. 6d). Finally, we assessed the role of ROS and *pmk-3* in the lifespan extension by lactate or pyruvate. The use of 10 mM NAC or *pmk-3* mutants prevented lifespan extension (Fig. 6e–g). These observations strengthen the notion that lactate and pyruvate produce mild ROS elevations, which in turn trigger an increase in the expression of detoxifying mechanisms promoting stress resistance.

**Fig. 7:**
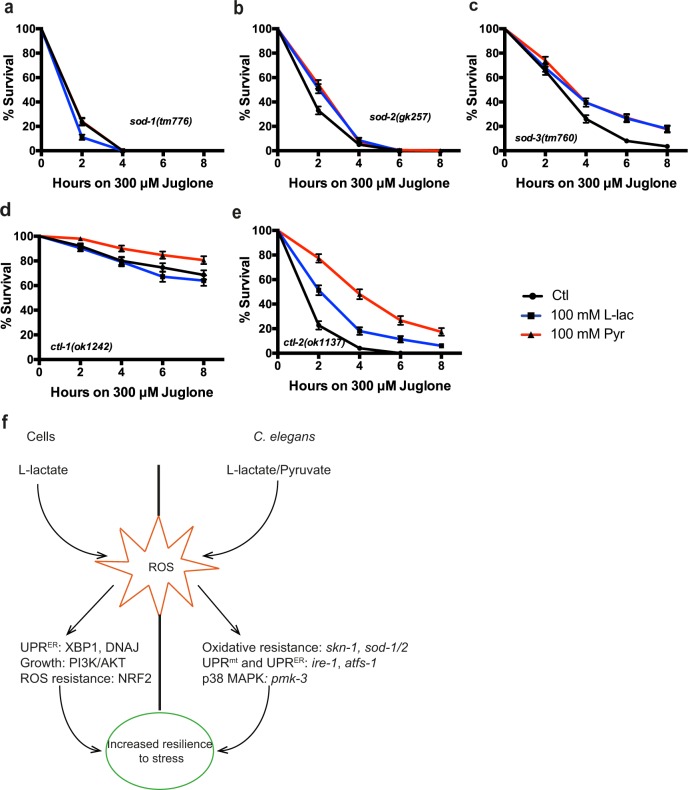
ROS detoxifying enzymes influence stress resistance and longevity induced by lactate and pyruvate. **a**–**c** Survival curves for superoxide dismutase mutants treated with 300 μM juglone and supplemented with 100 mM lactate or pyruvate. **d**, **e** Survival curves for catalases mutants treated with 300 μM juglone and supplemented with 100 mM lactate or pyruvate. **f** Schematic illustrating the effect of lactate and pyruvate on stress resistance and longevity in SH-SY5Y cells (in vitro) and in *C. elegans* (in vivo). Survival curves were analyzed using the Log-Rank (Mantel-Cox) test. 50 animals/trial, *N* *=* *3*

Finally, the superoxide dismutase loss of function mutants *sod-1* and *sod-2* prevented stress resistance by lactate or pyruvate, while *sod-3* mutants had no effects on lactate- or pyruvate-mediated protection (Fig. 7a–c). Catalase loss of function mutant *ctl-1* displayed a high-stress resistance that was only increased by pyruvate, while *ctl-2* mutation did not influence lactate- or pyruvate-mediated stress resistance (Fig. 7d, e).

## Discussion

In this study, we report a new mechanism underlying the role of lactate in cell survival. Lactate is known to reduce the toxicity of different cellular insults such as glutamate excitotoxicity or cerebral ischemia^10,11^ and promotes recovery through activation of PI3K/AKT pathway. We analyzed how monocarboxylates influence cell survival against oxidative stress, in the context of aging and neurodegeneration. SH-SY5Y cells pre-treated with lactate showed increased survival under oxidative stress conditions. In line with previous findings^11^, transcriptome analysis of these cells revealed that lactate induces activation of PI3K/AKT and mTOR pathways. Interestingly, lactate also promotes the expression of DNAJ chaperones and unfolded protein response (UPR) genes (Fig. S2e–g), elements well-known to underlie the endoplasmic reticulum stress response^56^. Taken together, these observations indicate that lactate improves cell survival through an increase in ER protein homeostasis as well as via activation of the PI3K signaling pathway.

It was recently shown that lactate induces ROS^39,40^. We report here that this increase promotes stress resistance and longevity through the mild activation of pro-oxidative mechanisms in neuroblastoma and *C. elegans*. These observations are in line with mitohormesis, a concept described in multiple organisms^20,57^ whereby a moderate increase in ROS production, in the low micromolar range (Fig. 3f), promotes stress resistance and longevity^22,58^. This hypothesis is supported by recent literature pointing to the signaling role of ROS^18,19^. Thus, results reported here (Fig. 3, 4) suggest that lactate supports cell survival through a mild elevation in ROS levels that involves a boost in mitochondrial activity indicative of mitohormesis. Moreover, ROS increase PI3K, mTOR signaling as well as HIF1α activation in vitro^59^, suggesting that the mild pro-oxidative environment activates those pathways to promote cell survival. In line with previous observations, inhibition of the Insulin/IGF signaling (IIS) increased stress resistance, but neither lactate nor pyruvate could further increase juglone resistance. This could be explained by an increased ROS concentration in IIS mutants compared to wild-type animals^60^, mimicking lactate and pyruvate effects.

ROS are mainly produced in the cytoplasm by NADPH oxidase and in the mitochondria by the leak of protons during oxidative metabolism. Our observations using JC-1 (Fig. 3g) indicate that lactate increases mitochondrial activity. Others have shown that lactate can be oxidized directly in the mitochondria^61^. Yet, another possibility could be that NADH, produced during the conversion of lactate into pyruvate, could enter the mitochondria via the malate-asparate shuttle, thereby boosting the electron transport chain and ROS levels^62^. Furthermore, our observations in the worm show that both lactate and pyruvate promote ROS production, suggesting a metabolic-related phenomenon. The inactivation of NAPDH oxidase using apocynin in vitro (Fig. S1h) or a mutant of DUOX ortholog *bli-3* (Fig. S4d) did not influence lactate or pyruvate-mediated stress resistance. This indicates that lactate-mediated stress resistance is not linked to this enzyme. It is likely that the sensitivity to lactate or pyruvate can vary between different cell types. This variation could be explained by the differential expression of metabolic-related enzymes (glycolytic vs. oxidative metabolism) as observed between astrocytes and neurons^6,63^. As a consequence, this may influence how the metabolites trigger cellular and metabolic response in specific cell types. A more exhaustive approach using multiple cell lines or organisms would be required to answer this question adequately.

However, the present results in cells and nematodes show increased activation of NRF2 crucial transcription factor activated by oxidative stress^64^, and its downstream effector *gst-4*, respectively. In the nematode, this activation promotes stress resistance through *pmk-3 and ire-1* dependent signaling (Fig. 6b). Although PMK3 function has, so far, not been linked to *skn-1* activity, p38 MAPK ortholog *pmk-1* promotes *skn-1* and *gst-4* activation through NADPH/*bli-3* and *ire-1* signaling^51^. However, neither, loss of *bli-3* (Fig. S4d) nor MAPKKK *dlk-1* (Fig. S4h)*, pmk-3* canonical upstream MAPKKK^54^, blocked stress resistance by lactate or pyruvate. Interestingly, the loss of *hif-1*, although displaying an increased expression of *gst-4* and ROS level compared to wild-type background, failed to respond to lactate- or pyruvate-increased stress resistance (Fig. 6b, c). Therefore, it appears that the activation window is controlled carefully to produce positive outcomes.

## Conclusion and perspective

In this report, we show that lactate promotes stress resistance in SH-SY5Y cells, while both lactate and pyruvate promote stress resistance in *C. elegans*. It appears that in both models, this protective mechanism occurs through ROS signaling. This is, to our knowledge, the first report that links lactate-mediated ROS induction and increased proteostasis. In the context brain aging and disorders where lactate release appears to be reduced, boosting the production and release of lactate by astrocytes to support neurons^65^ would be a promising therapeutic strategy to favor brain health.

## Supplementary information

Supplementary information

Table S1

Table S2

Dataset 1

Dataset 2
