# Supplementary material for: Lactate and pyruvate promote oxidative stress resistance through hormetic ROS signaling
Source: Cell Death Dis. 2019 Sep 10;10(9):653. doi: 10.1038/s41419-019-1877-6 (PMC6737085; doi:10.1038/s41419-019-1877-6)
Supplement: Supplementary file 1 — Supplementary information [file 41419_2019_1877_MOESM1_ESM.pdf]

SUPPLEMENTARY INFORMATION

Lactate and pyruvate promote oxidative stress resistance through hormetic ROS signaling.

Arnaud Tauffenberger[1]*, Hubert Fiumelli[1], Salam Almustafa[1,2], and Pierre J. Magistretti[1]*

[1]Laboratory for Cellular Imaging and Energetics, Biological and Environmental Sciences and Engineering Division, King Abdullah University of Science and Technology, Thuwal, 23955-6900, Kingdom of Saudi Arabia [2]Imam Abdulrahman bin Faisal University, Dammam, Kingdom of Saudi Arabia

* Correspondance :
pierre.magistretti@kaust.edu.sa
4700 KAUST, Thuwal, 23955-6900, KSA
+966

arnaud.tauffenberger@kaust.edu.sa
4700 KAUST, Thuwal, 23955-6900, KSA
+966540380273

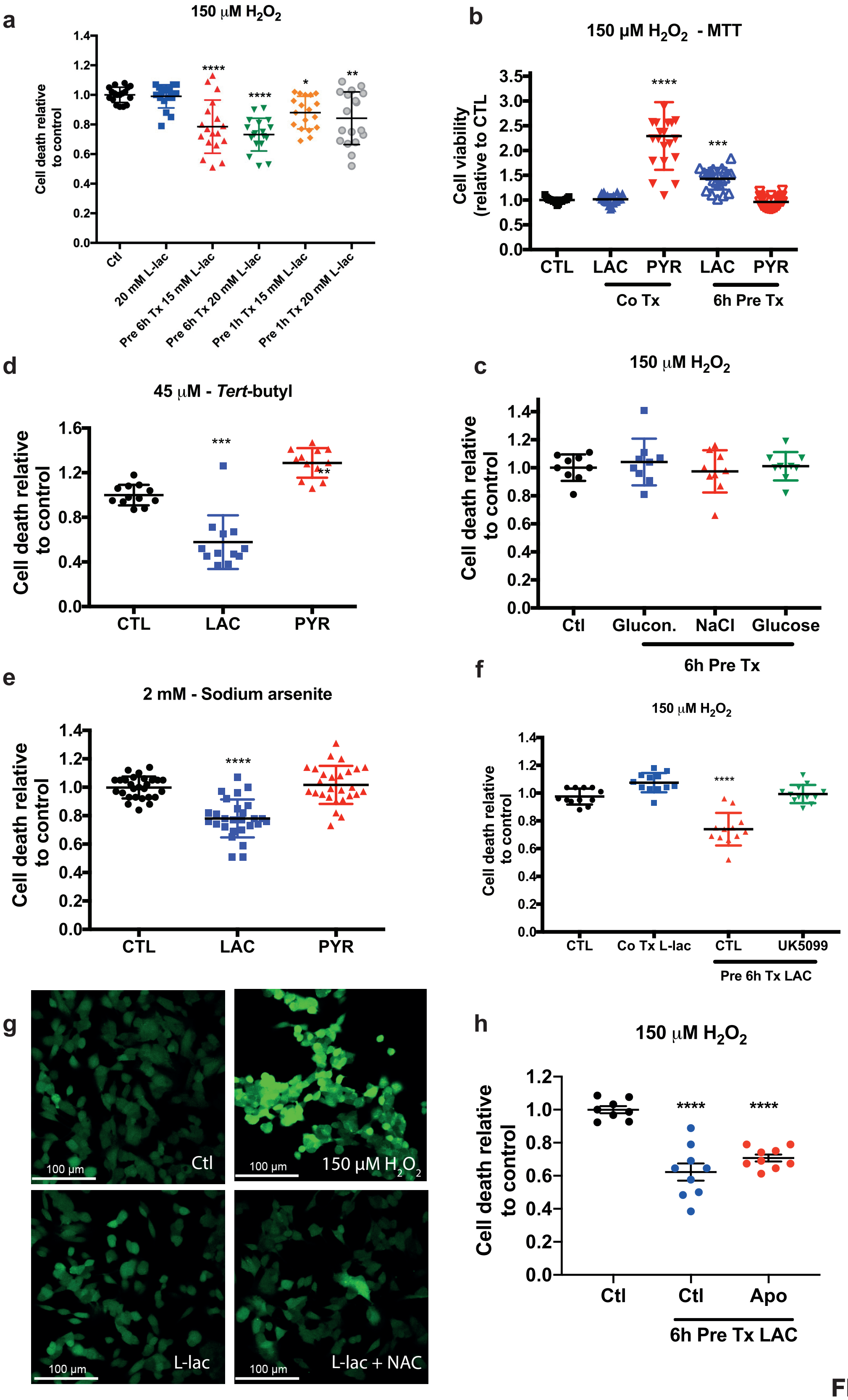

a
150 μM H2O2
Cell death relative to control
Ctl
20 mM L-lac
Pre 6h Tx 15 mM L-lac
Pre 6h Tx 20 mM L-lac
Pre 1h Tx 15 mM L-lac
Pre 1h Tx 20 mM L-lac
b
150 μM H2O2 - MTT
Cell viability (relative to CTL)
CTL
LAC
PYR
LAC
PYR
Co Tx
6h Pre Tx
d
45 μM - Tert-butyl
Cell death relative to control
CTL
LAC
PYR
c
150 μM H2O2
Cell death relative to control
Ctl
Glucon.
NaCl
Glucose
6h Pre Tx
e
2 mM - Sodium arsenite
Cell death relative to control
CTL
LAC
PYR
f
150 μM H2O2
Cell death relative to control
CTL
Co Tx L-lac
CTL
UK5099
Pre 6h Tx LAC
g
100 μm
Ctl
100 μm
150 μM H2O2
100 μm
L-lac
100 μm
L-lac + NAC
h
150 μM H2O2
Cell death relative to control
Ctl
Ctl
Apo
6h Pre Tx LAC

FIG. S1

Supplementary Figure 1: Characterization of lactate protective response. *(Related to Figure 1)*

a) Relative measures of cell death after 150 μM H_2O_2 treatment upon pre-treatment with lactate (15mM -20mM) for different durations (1h – 6h). **b)** Relative measures of cell death induced by 150 μM H_2O_2 as determined by the MTT assay upon pre-treatment with lactate (20 mM – 6h). **c)** A measure of cell death after 150 μM H_2O_2 treatment upon pre-treatment with D-glucose, NaCl or Na-gluconate (all 20mM). **d-e)** Measure of cell death upon pre-treatment with lactate (20 mM – 6h) after treatment with 45 μM tert-butyl **d)** or 2mM Sodium Arsenite **e)**. **f)** The measure of cell death after 150 μM H_2O_2 and upon pre-treatment with lactate (20 mM – 6h) using MCT blocker UK5099. **g)** Confocal images of SH-SY5Y cells stained with H_2DCFDA (50 μM). Cells were treated with 150 μM H_2O_2 for 30 min or with 20mM lactate for 6h. **h)** Relative measure of cell death after 150 μM H_2O_2 treatment upon pre-treatment with lactate (20 mM) using NADPH oxidase inhibitor apocynin (50 μM)

Bars are the average ± SEM (*N=4*, *p<0.05, **p<0.01, ***p<0.001, ****p<0.0001)

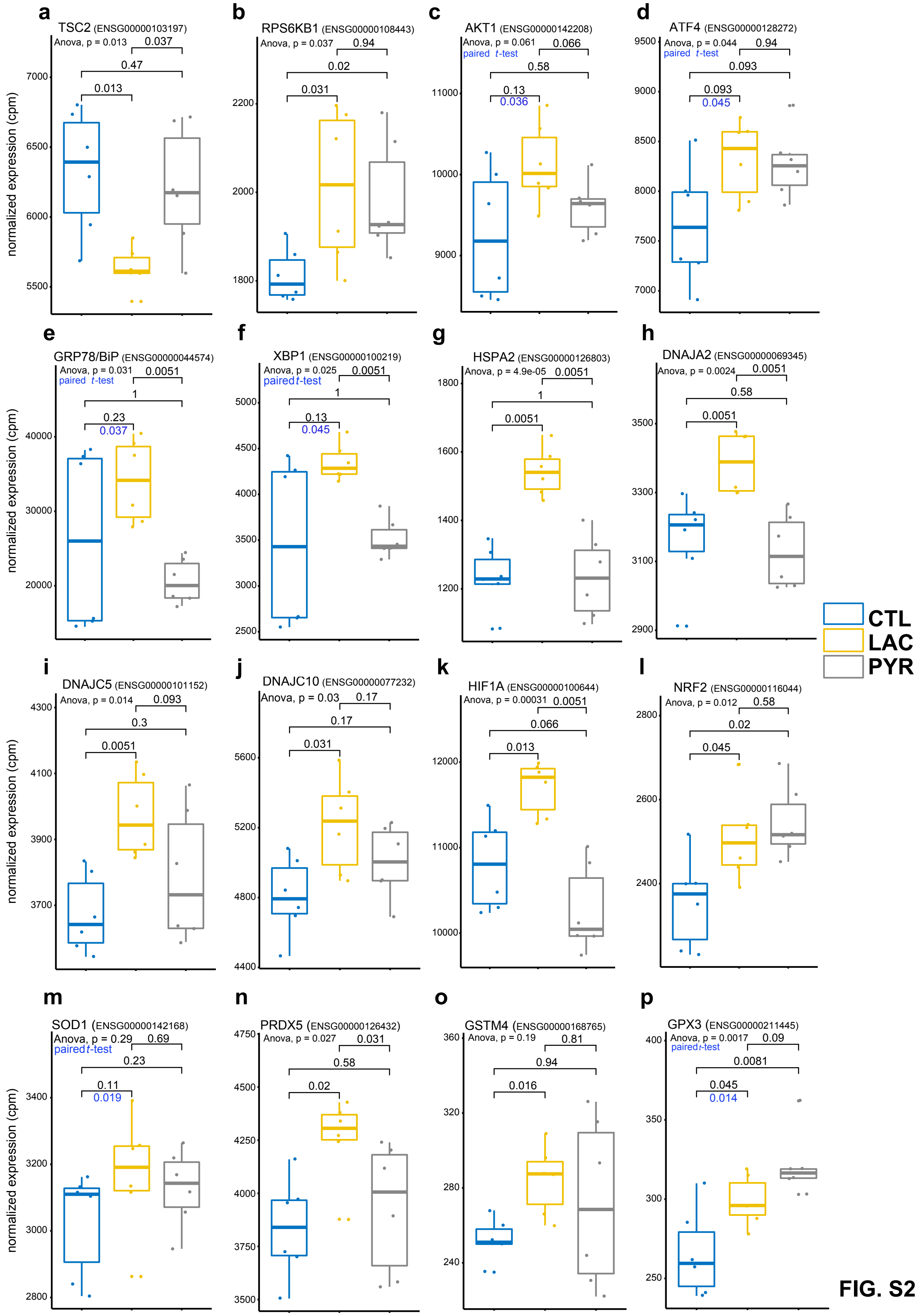
a TSC2 (ENSG00000103197)
b RPS6KB1 (ENSG00000108443)
c AKT1 (ENSG00000142208)
d ATF4 (ENSG00000128272)
e GRP78/BiP (ENSG00000044574)
f XBP1 (ENSG00000100219)
g HSPA2 (ENSG00000126803)
h DNAJA2 (ENSG00000069345)
i DNAJC5 (ENSG00000101152)
j DNAJC10 (ENSG00000077232)
k HIF1A (ENSG00000100644)
l NRF2 (ENSG00000116044)
m SOD1 (ENSG00000142168)
n PRDX5 (ENSG00000126432)
o GSTM4 (ENSG00000168765)
p GPX3 (ENSG00000211445)
normalized expression (cpm)
CTL
LAC
PYR

FIG. S2

S**upplementary Figure 2: Expression levels of genes differentially regulated by lactate when compared to control or lactate treatment identified in the RNAseq analysis of SH-SY5Y cells**. ***(Related to Figure 2)***

Boxplots showing normalized transcript levels of mTOR and PI3K related candidates; **a)** TSC2, **b)** RPS6KB1, **c)** AKT1 and **D)** ATF4. Transcript levels for ER processing related candidates; **e)** GRP78/BiP, and **f)** XBP1, **g)** DNAJA2, **h** DNAJC5, **i)** DNAJC10, **j)** HSPA2.

Transcripts levels of candidates related to ROS induction and detoxification; **k)** HIF1α, **l)** NRF2, **m)** SOD1, **n)** PRDX53, **o)** GSTM4 and **p)** GPX3.

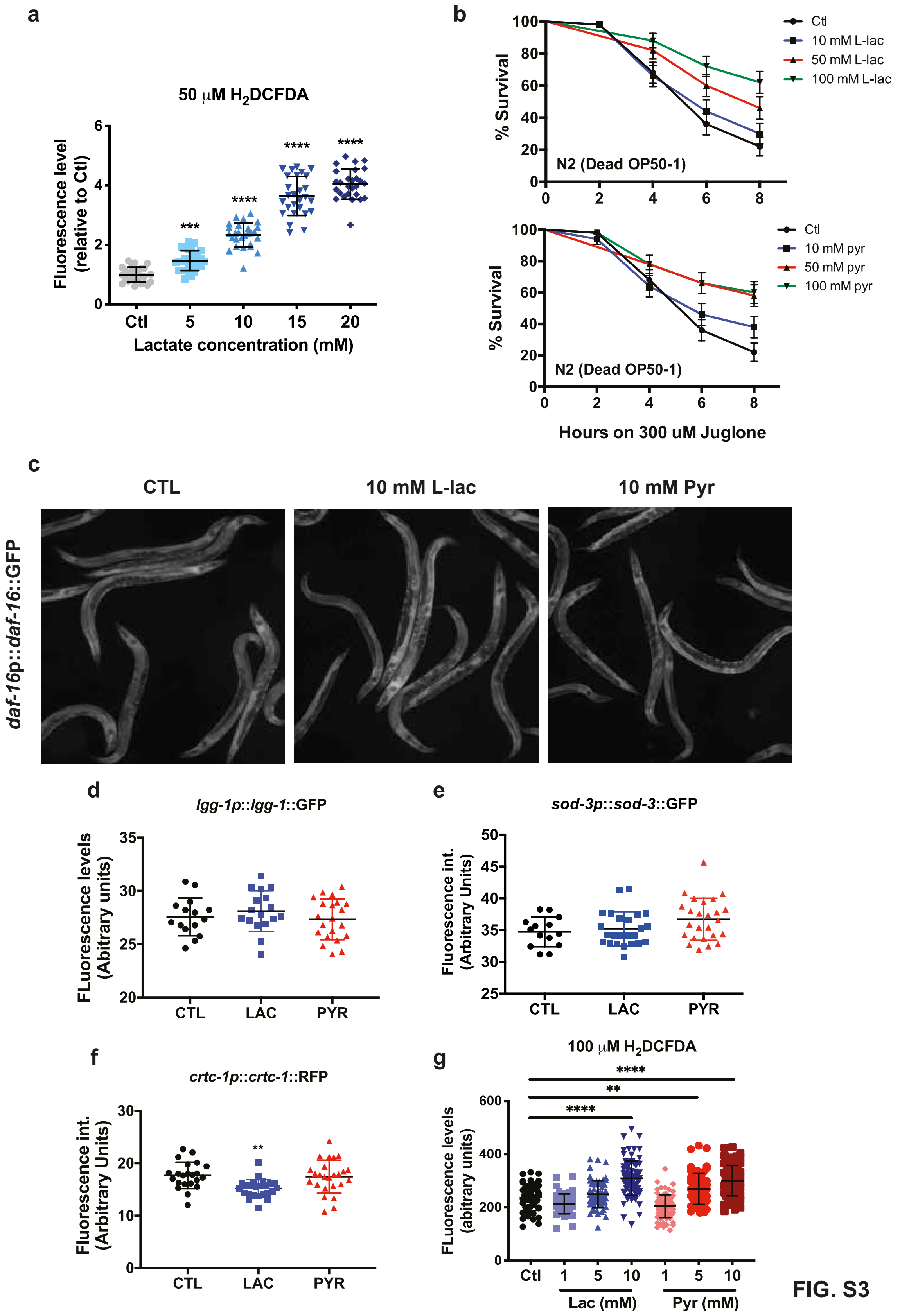
a
50 μM H2DCFDA
Fluorescence level (relative to Ctl)
Ctl
5
10
15
20
Lactate concentration (mM)
b
% Survival
Ctl
10 mM L-lac
50 mM L-lac
100 mM L-lac
N2 (Dead OP50-1)
Ctl
10 mM pyr
50 mM pyr
100 mM pyr
Hours on 300 uM Juglone
c
CTL
10 mM L-lac
10 mM Pyr
daf-16p::daf-16::GFP
d
lgg-1p::lgg-1::GFP
FLuorescence levels (Abitrary units)
CTL
LAC
PYR
e
sod-3p::sod-3::GFP
Fluorescence int. (Arbitrary Units)
f
crtc-1p::crtc-1::RFP
g
100 μM H2DCFDA
FLuorescence levels (abitrary units)
Lac (mM)
Pyr (mM)

FIG. S3

Supplementary Figure 3: Characterization of longevity phenotypes induced by lactate and pyruvate. ***(Related to figures 3 and 4)***

a) Measure of ROS in SH-SY5Y cells after 6h treatment with different doses of lactate. **b)** Survival curves of wild-type (N2) animals treated with 300µM juglone and supplemented with 10, 50 and 100 mM lactate or pyruvate before the test. Worms were grown on dead OP50-1. Measure of expression for translational reporter of *daf-16* **c)**, *lgg-1* **d)**, *sod-3* **e)** and *crtc-1* **f)** after dietary supplementation with 10 mM lactate or pyruvate. **d)** Measure of ROS in wild-type (N2) animals at different concentrations of lactate or pyruvate.

Bars are the average ± SEM (*N=3*, *p<0.05, **p<0.01, ***p<0.001 and ****p<0.0001)

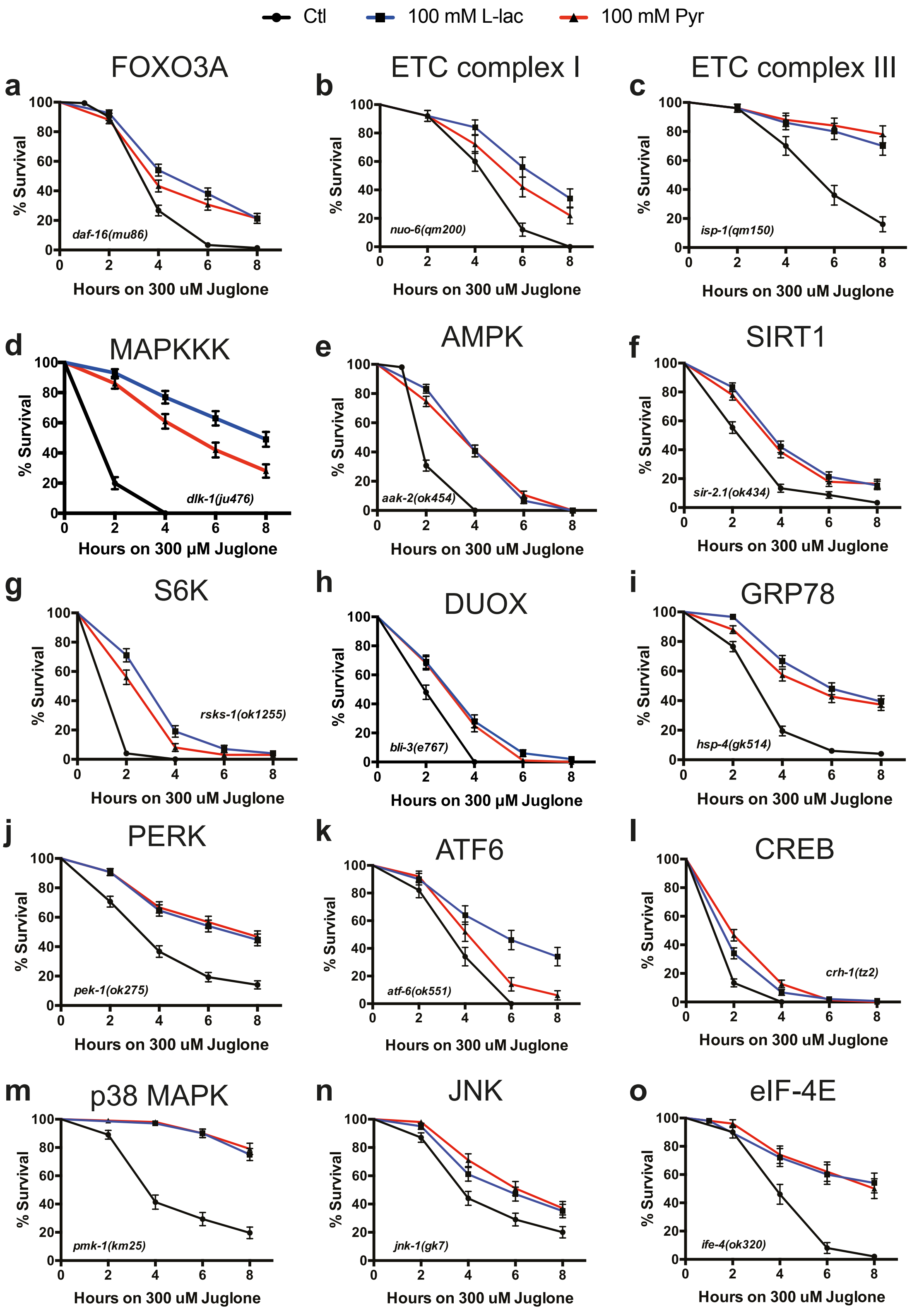

Ctl
100 mM L-lac
100 mM Pyr
a
FOXO3A
daf-16(mu86)
b
ETC complex I
nuo-6(qm200)
c
ETC complex III
isp-1(qm150)
d
MAPKKK
dlk-1(ju476)
e
AMPK
aak-2(ok454)
f
SIRT1
sir-2.1(ok434)
g
S6K
rsks-1(ok1255)
h
DUOX
bli-3(e767)
i
GRP78
hsp-4(gk514)
j
PERK
pek-1(ok275)
k
ATF6
atf-6(ok551)
l
CREB
crh-1(tz2)
m
p38 MAPK
pmk-1(km25)
n
JNK
jnk-1(gk7)
o
eIF-4E
ife-4(ok320)
% Survival
Hours on 300 uM Juglone
Hours on 300 μM Juglone

FIG. S4

Supplementary Figure 4: Different metabolic sensitive pathways are not required for lactate and pyruvate stress resistance. ***(Related to figure 5)***

a-o) Survival curves of mutant nematodes treated 300µM juglone and supplemented with 100mM lactate or pyruvate. Mutated genes are indicated as an inset in the lower left or right corner of each graph, and mammalian orthologs are indicated above each graph. *N=3*

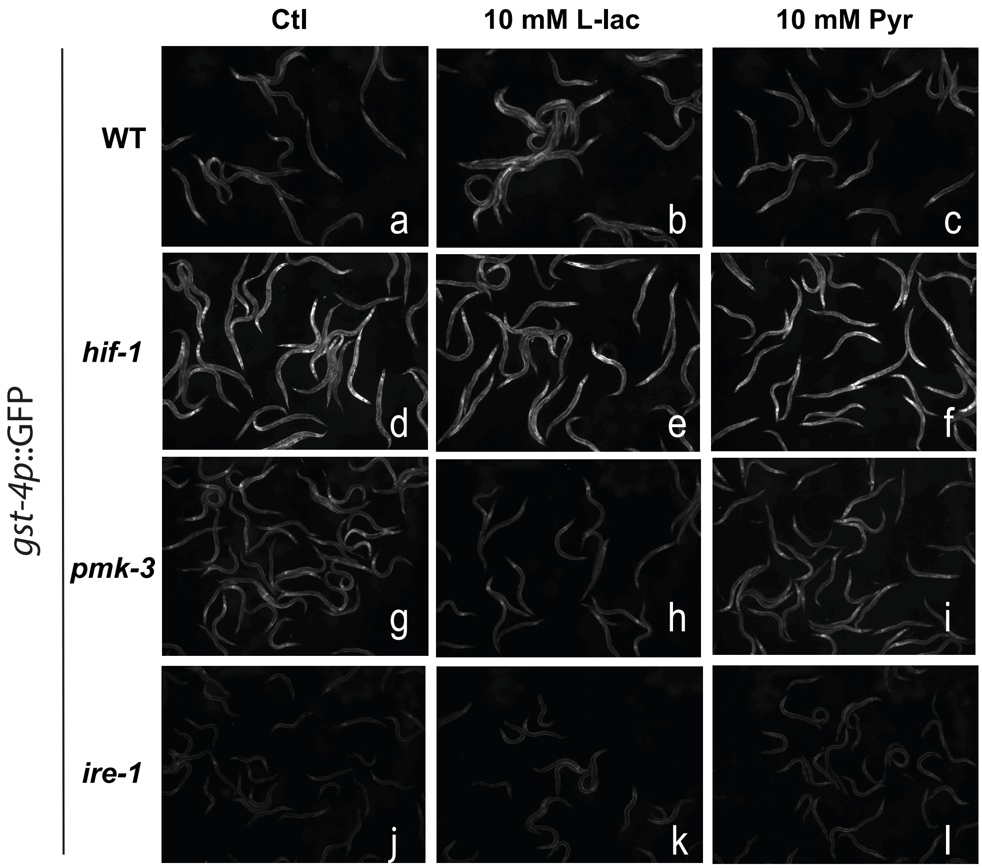

Supplementary figure 5: Effects of lactate and pyruvate on the levels of *gst-4p*::GFP in wild-type and mutant animals. ***(Related to Figure 6)***

a-c) Fluorescent images of gst-4p:GFP in wild-type animals imaging upon 10 mM lactate or pyruvate diet supplementation. **d-f)** Animals with *hif-1* mutant background imaging upon 10 mM lactate or pyruvate diet supplementation. **g-i)** Animals with *pmk-3* mutant background imaging upon 10 mM lactate or pyruvate diet supplementation. **j-l)** Animals with *ire-1* mutant background imaging upon 10 mM lactate or pyruvate diet supplementation.
