# Supplementary material for: Lactate and pyruvate promote oxidative stress resistance through hormetic ROS signaling
Source: Cell Death Dis. 2019 Sep 10;10(9):653. doi: 10.1038/s41419-019-1877-6 (PMC6737085; doi:10.1038/s41419-019-1877-6)
Supplement: Supplementary file 2 — Table S1 [file 41419_2019_1877_MOESM2_ESM.docx]

Table S1: Strains list used in Tauffenberger et al.

| Strain name | Genotype | Source |
| --- | --- | --- |
| N2 |  | CGC |
| CF1038 | *daf-16(mu86)* | CGC |
| ZG31 | *hif-1(ia4)* | CGC |
| BS3383 | *pmk-3(ok169)* | CGC |
| PS3551 | *hsf-1(sy441)* | CGC |
| RE666 | *ire-1(v33)* | CGC |
| VC1099 | *hsp-4(gk514)* | CGC |
| RB772 | *atf-6(ok551)* | CGC |
| CB1370 | *daf-2(e1370)* | CGC |
| RB759 | *akt-1(ok525)* | CGC |
| VC204 | *akt-2(ok393)* | CGC |
| GA187 | *sod-1(tm776)* | CGC |
| GA186 | *sod-3(tm760* | CGC |
| GA184 | *sod-2(gk257)* | CGC |
| TJ1052 | *age-1(hx546)* | CGC |
| JIN1375 | *hlh-30(tm1978)* | CGC |
| PY1589 | *cmk-1(oy21)* | CGC |
| RB754 | *aak-2(ok524)* | CGC |
| VC199 | *sir-2.1(ok434)* | CGC |
| RB1206 | *rsks-1(ok1255)* | CGC |
| RB545 | *pek-1(ok275)* | CGC |
| YT17 | *crh-1(tz2)* | CGC |
| KU25 | *pmk-1(km25)* | CGC |
| KX17 | *ife-4(ok320)* | CGC |
| VC8 | *jnk-1(gk7)* | CGC |
| KU4 | *sek-1(km4)* | CGC |
| VC3201 | *atfs-1(gk3094)* | CGC |
| SJ4100 | zcIs13[*hsp-6*::GFP] | CGC |
| SJ4005 | zcIs4 [*hsp-4*::GFP] | CGC |
| SJ4058 | zcIs9 [*hsp-60*::GFP + *lin-15*(+)] | CGC |
| CF1553 | muIs84 [(pAD76) *sod-3p*::GFP + *rol-6*(*su1006*)] | CGC |
| TJ356 | zIs356 [*daf-16p*::*daf-16a*/b::GFP  *rol-6*(*su1006*)] | CGC |
| CL2166 | dvIs19 [(pAF15)*gst-4p*::GFP::NLS] | CGC |
| DA2123 | adIs2122[*lgg-1p*::GFP::*lgg-1* + *rol-6(su1006)*] | CGC |
| AGD418 | uthIs205 [*crtc-1p*::*crtc-1*::RFP::*unc-54* 3'UTR + *rol-6*(*su1006*)] | CGC |
| WS4274 | opIs206 [*hif-1p*::*hif-1*(genomic)::GFP::*hif-1* 3'UTR + *unc-119*(+)] | CGC |
| PJM4 | dvIs19 [(pAF15)*gst-4p*::GFP::NLS]; ];  *hif-1(ia4)* | This study |
| PJM6 | dvIs19 [(pAF15)*gst-4p*::GFP::NLS];  *pmk-3(ok169)* | This study |
| PJM7 | dvIs19 [(pAF15)*gst-4p*::GFP::NLS];  *ire-1(v33)* | This study |
