# Supplementary material for: Lactate and pyruvate promote oxidative stress resistance through hormetic ROS signaling
Source: Cell Death Dis. 2019 Sep 10;10(9):653. doi: 10.1038/s41419-019-1877-6 (PMC6737085; doi:10.1038/s41419-019-1877-6)
Supplement: Supplementary file 3 — Table S2 [file 41419_2019_1877_MOESM3_ESM.docx]

**Table S2: Lifespan statistics**

| Figure | Genotype | Mean lifespan | Maximum lifespan | P value | Numbers of animals |
| --- | --- | --- | --- | --- | --- |
| **4** | N2 | 16 | 26 |  | 270 |
|  | +10 mM L-lactate | 22 | 32 | <0.0001 | 270 |
|  | + 100 mM L-lactate | 16 | 20 | 0.0019 | 270 |
|  | +10 mM Pyruvate | 22 | 32 | <0.0001 | 270 |
|  | + 100 mM pyruvate | 16 | 20 | 0.0275 | 270 |
| **6** | N2 | 14 | 26 |  | 180 |
|  | +10 mM L-lactate | 18 | 28 | <0.0001 | 180 |
|  | +10 mM pyruvate | 18 | 30 | <0.0001 | 180 |
|  | *pmk-3(ok169)* | 12 | 18 |  | 180 |
|  | +10 mM L-lactate | 13,5 | 20 | n.s. 0.4600 | 180 |
|  | +10 mM pyruvate | 15 | 18 | n.s. 0.6157 | 180 |
|  | 10 mM NAC | 15 | 22 |  | 180 |
|  | +10 mM L-lactate | 17 | 22 | n.s. 0.0645 | 180 |
|  | +10 mM pyruvate | 16 | 22 | n.s. 0.1772 | 180 |
